# Not all cemented hips are the same: a register-based (NJR) comparison of taper-slip and composite beam femoral stems

**DOI:** 10.1080/17453674.2019.1582680

**Published:** 2019-03-06

**Authors:** Hussain A Kazi, Sarah L Whitehouse, Jonathan R Howell, A John Timperley

**Affiliations:** a Princess Elizabeth Orthopaedic Centre, Royal Devon and Exeter NHS Foundation Trust, Exeter, UK;; b Queensland University of Technology (QUT), Brisbane, Queensland, Australia;; c University of Exeter, Exeter, UK

## Abstract

Background and purpose — No difference in outcome has been demonstrated comparing cemented taper-slip and composite beam designs in short-term randomised trials; we assessed outcome differences using a registry analysis.

Patients and methods — All cemented stems with > 100 implantations were identified in the National Joint Registry of England and Wales from April 1, 2003 to September 31, 2013 and categorised as taper-slip or composite beam. Survival analyses using Kaplan–Meier and Cox regression were performed.

Results — We identified 292,987 cemented arthroplasties, of which 16% (47,586) were composite beam stems, with taper-slip stems making up the remainder (n = 245,401). There was a statistically significant increased chance of revision in the composite beam group compared with the taper-slip group (1.7% vs 1.3%, p < 0.001) but statistically no significant differences of survival estimates (p = 0.06). When the 2 groups were segregated to delineate the most implanted model in each category, the differences became more profound with the most implanted taper-slip stem (Exeter V40) showing statistically and clinically significant superior 8-year survival: 97.9% compared with 97.6% for all other taper-slip; 97.5% for the most implanted composite beam (Charnley cemented stem); and 97.7% for all other composite beam.

Interpretation — There was an increased incidence of revision for composite beam stems. The most implanted taper-slip stem demonstrated significant survival advantage vs. all other stems.

Cemented femoral stems can be divided into designs that achieve fixation as a composite beam and those that function as a taper-slip device (Shah and Porter 2005). Taper-slip stem designs function by controlled stem subsidence within the cement mantle whereas composite beam stems seek mechanical interlock at all interfaces including fixation between the stem and cement.

Radiostereometry studies (Alfaro-Adrian et al. 2001) have shown differences between taper-slip and composite beam stems with respect to their migration and micromotion. Polished tapered stems subside within cement, with no movement occurring at the cement–bone interface. In contrast composite beam stems subside over smaller distances but crucially this occurs at both the stem–cement and cement–bone interfaces. Movement of the cement in relation to bone indicates that fixation at the cement–bone interface is compromised and the cement cannot be osseointegrated (Schmalzried et al. 1992).

Despite the findings in in vitro and implant retrieval studies (Huiskes et al. 1998, Verdonschot and Huiskes 1998, Howell et al. 2004), most in vivo reports have failed to determine a difference in outcome between composite beam and taper-slip designs (Lachiewicz et al. 2008, Jayasuriya et al. 2013), most likely due to small numbers.

We investigated revision rates in the UK for primary cemented hips by prosthesis subgroup of taper-slip and composite beam stems.

## Patients and methods

The National Joint Registry of England & Wales (and latterly Northern Ireland and Isle of Man) (NJR) was established in 2002. Patient demographics and surgical details are recorded, with mortality information being updated biannually and subsequent revisions linked to the primary operation, with more than 94% completeness reported (Porter 2017).

We performed an approved retrospective cohort study of the NJR dataset. Data were requested to provide information regarding potential confounding factors. The study population included all validated cemented primary total hip operations performed in England and Wales from April 1, 2003 to September 30, 2013, as this request preceded Northern Ireland and the Isle of Man joining the NJR (2013 and 2015 respectively). The mean length of follow-up for this cohort was 4.2 years (0–12).

Using the criteria by Huiskes (1998) (Table 1), stem designs were subdivided in terms of whether they were taper-slip or composite beam using published data (predominantly surface finish). Only stems with >100 implantations were included.

**Table 1.** Design features of different cemented stems (after Huiskes et al.^1998^)

Design	Force closed (taper-slip)	Shape closed (composite beam)
Surface Finish	Polished	Roughened/matt
Taper	+	+/–
Collar	–	+
Ridges/flanges/profiles	–	+

In order to remove bias of metal-on-metal hips in analysing the effect of stem design on outcome, the definitive analysis was performed excluding metal-on-metal and ‘unknown’ bearing couples.

The most commonly implanted stems of both designs were then separated in order to examine whether stems with the same design philosophy function in an identical fashion giving equivocal results. The final analysis therefore comprised 4 cohorts: most implanted taper-slip (Exeter, Stryker Orthopaedics, Mahwah, NJ); all other taper-slip; most implanted composite beam (Charnley, DePuy Orthopaedics, Warsaw, IN); and all other composite beam. 

### Statistics

Statistical analysis was performed using IBM SPSS (Version 22, IBM Corp, Armonk, NY, USA) and NCSS (NCSS 10 Statistical Software (2015). NCSS, LLC. Kaysville, UT, USA, ncss.com/software/ncss). Cox regression analysis (using the Enter method where all variables are added as a single block) was used to identify revision rates within subgroups and factors influencing these rates. Hazard ratios (HR) and 95% confidence intervals (CI) are presented. Frequencies were compared using the chi-squared (χ^2^) test and continuous variables compared using analysis of variance (ANOVA). Confounding factors were investigated: age, sex, ASA grade, procedure type (routine/complex), diagnosis, approach, and bearing couple. Surgeon grade and provider type (public or private) were not provided by the NJR. Data validation was performed prior to analysis by scrutiny of the data, including categorisation of stem types, use of cement, examination of missing and invalid responses according to surgical details, and coding and validation of diagnosis and reasons for revision. Following validation, there were minimal missing values (5 for sex) other than for approach, where these cases were treated as a separate group in order to determine whether any bias existed. The 5 cases with missing sex were excluded from the Cox regression model. Kaplan–Meier survival curves were constructed with cut-off at 8 years where the appropriate effective number of cases at risk remained, utilising the guidance stipulated by Pocock et al. (2002) and Lettin et al. (1991) and cumulative survival compared using the log-rank test. Competing risk analysis was not adopted as it is more appropriate when the risk of death is high (Gillam et al. 2010) and may not be the most appropriate for estimating implant failure (Sayers et al. 2018).

### Ethics, funding, and potential conflicts of interests

This work was approved by the National Joint Registry Research Sub-Committee. The work involves de-identified data so is exempt from IRB approval.

JRH and AJT have received or will receive benefits for personal or professional use from Stryker Corporation. In addition, benefits have been directed to a research fund and educational institution with which SLW, JRH, and AJT are associated. No funding was received specifically for this project, and there was no input from any commercial interest for any aspect of this study.

## Results

292,987 primary cemented hip replacements were included. Composite beam stems accounted for 16% (47,586 hips), with the remainder being taper-slip stems. Exeter V40 was the commonest taper-slip design and Charnley cemented stem the commonest composite beam design (Table 2, see Supplementary data). There was a tendency for composite beam stems to be used in slightly older patients (mean 73.6 years) than taper-slip (mean 71.9 years) (Table 3, see Supplementary data) although this is unlikely to be clinically relevant. There was a higher proportion of deaths (17.2%) in the composite beam group compared with 10.5% in the taper-slip group (Tables 3 and 4, see Supplementary data), but more detailed exploration is beyond the scope of this project. Ignoring the deaths in both groups, there was a statistically significant increased chance of revision in the composite beam group compared with the taper-slip group (1.7% vs. 1.3%, p < 0.001) (Table 3, see Supplementary data).

**Table 2.** Stem type and brand frequencies

Stem	Frequency
**Taper-slip**	
Exeter V40	175,472
CPT	29,512
C-Stem Cemented Stem	17,477
C-Stem AMT Cemented Stem	8,994
MS-30	3,415
CPCS	2,468
CPS Plus	2,119
Furlong Cemented Stem	1,877
Taperfit Cemented Stem	1,313
Taperloc Cemented Stem	1,034
Olympia	904
Exeter	886
Ultima TPS Stem	176
Aeon Cemented Stem	261
Profemur Cemented Stem	241
Corail Cemented	135
Edinburgh	117
Total	245,401
**Composite beam**	
Charnley Cemented Stem	22,015
Stanmore Modular Stem	7,016
Muller-Biomet	3,107
Muller Straight Stem	2,481
SP II Cemented Stem	2,372
Elite Plus Cemented Stem	1,719
Omnifit Cemented Stem	1,513
CCA Cemented Stem	1,302
Charnley Modular	1,126
P10 Muller	838
Spectron	838
Centrament	671
VerSys Cemented Stem	623
CMK Cemented Stem	561
Excia Cemented	438
Ultima Straight Stem	396
Mem	371
Stanmore Monobloc Stem	237
Bimetric Cemented (Ti)	209
Hi-Nek Cemented Stem	129
Summit Cemented Stem	108
Bimetric Cemented (CoCr)	104
Total	47,586

**Table 3.** Comparison of taper-slip and composite beam stems

Factor	Stem type		p-value
	Taper-slip	Composite beam	
Number	245,401 (83.8%)	47,586 (16.2%)	n/a
Mean age (range)	71.9 (12–103)	73.6 (15–101)	< 0.001**^a^**
Death, n (%)	25,761 (10.5)	8,168 (17.2)	< 0.001^b^
Revised, n (%)	3,080 (1.3)	810 (1.7)	< 0.001^b^

a ANOVA

b χ^2^ test

**Table 4.** Breakdown by stem type. Values are frequency (%) unless otherwise stated

	Taper-slip		Composite beam		p-value	
	Exeter	All other	Charnley	All other	2 groups	4 groups
Number	176,358	69,043	23,141	24,445	N/A	N/A
Age in years (range)	71.8 (12–100)	72.2 (15–103)	72.9 (19–101)	74.3 (15–101)	< 0.001^a^	< 0.001^a^
Death	18,234 (10.3)	7,527 (10.9)	4,215 (18.2)	3,953 (16.2)	< 0.001^b^	< 0.001^b^
Revised	2,108 (1.2)	972 (1.4)	428 (1.8)	382 (1.6)	< 0.001^b^	< 0.001^b^
Sex					< 0.001^b^	< 0.001^b^
Male	62,345 (35.4)	23,137 (33.5)	8,174 (35.3)	7,694 (31.5)		
Female	114,012 (64.6)	45,903 (66.5)	14,967 (64.7)	16,750 (68.5)		
ASA Grade					< 0.001^b^	< 0.001^b^
1	24,911 (14.1)	8,769 (12.7)	3,443 (14.9)	3,727 (13.6)		
2	120,756 (68.5)	48,327 (70.0)	15,043 (65.0)	16,859 (69.0)		
3	29,577 (16.8)	11,497 (16.7)	4,436 (19.2)	4,077 (16.7)		
4	1,073 (0.6)	436 (0.6)	210 (0.9)	169 (0.7)		
5	41 (0.02)	14 (0.02)	9 (0.04)	13 (0.1)		
Procedure type					< 0.001^b^	< 0.001^b^
Routine	168,339 (95.5)	65,662 (95.1)	19,466 (84.1)	21,894 (89.6)		
Complex	8,019 (4.5)	3,381 (4.9)	3,675 (15.9)	2,551 (10.4)		
Diagnosis					< 0.001^b^	< 0.001^b^
Osteoarthritis	158,824 (90.1)	62,244 (90.2)	21,341 (92.2)	22,351 (91.4)		
Other arthritides	2,651 (1.5)	943 (1.4)	403 (1.7)	319 (1.3)		
Previous trauma	2,313 (1.3)	876 (1.3)	212 (0.9)	317 (1.3)		
Acute trauma	4,489 (2.5)	1,579 (2.3)	229 (1.0)	596 (2.4)		
Previous hip surgery	328 (0.2)	172 (0.2)	13 (0.1)	23 (0.1)		
Childhood disease	1,807 (1.0)	820 (1.2)	103 (0.4)	116 (0.5)		
Avascular head necrosis	4,293 (2.4)	1,871 (2.7)	618 (2.7)	524 (2.1)		
Other (including infection)	1,653 (0.9)	538 (0.8)	222 (1.0)	199 (0.8)		
Approach					< 0.001^b^	< 0.001^b^
Transgluteal	73,779 (41.0)	26,466 (38.3)	16,638 (71.9)	14,028 (57.4)		
Posterior	92,779 (52.6)	36,047 (52.2)	1,442 (6.2)	6,906 (28.3)		
Trochanteric osteotomy	91 (0.1)	1,272 (1.8)	680 (2.9)	24 (0.1)		
Other	5,225 (3.0)	2,721 (3.9)	914 (3.9)	1,161 (4.7)		
Missing	5,934 (3.4)	2,527 (3.7)	3,467 (15.0)	2,326 (9.5)		
Bearing						
Metal-on-polyethylene	155,028 (87.9)	58,315 (84.5)	22,728 (98.2)	21,633 (88.5)		
Ceramic-on-ceramic	12,192 (6.9)	3,392 (4.9)	252 (1.1)	494 (2.0)		
Ceramic-on-polyethylene	8,853 (5.0)	7,251 (10.5)	160 (0.7)	2,300 (9.4)		
Ceramic-on-metal	285 (0.2)	85 (0.1)	1 (0.004)	18 (0.1)	< 0.001^b^	< 0.001^b^

a ANOVA

b χ^2^ test

**Figure 1.**
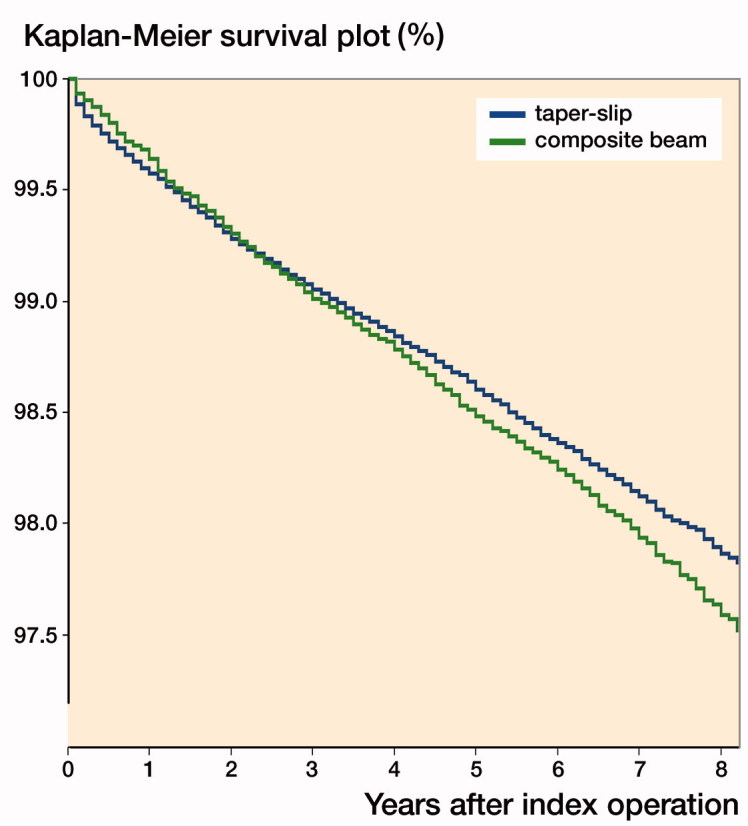
Kaplan–Meier survival curve for taper-slip and composite beam stems. Taper-slip 97.9% (CI 97.8–98.0) and composite beam 97.6% (97.4–97.8) 8-year survival.

Kaplan–Meier survival curves were constructed comparing the 2 groups (Figure 1). Both design philosophies had similar curves; log-rank test, p = 0.06: taper-slip stem 97.9% (CI 97.8–98.0); composite beam 97.6% (CI 97.4–97.8) at 8 years.

The dataset was further analysed comparing the most implanted taper-slip stem (Exeter), all other taper-slip stems, most implanted composite beam stem (Charnley), and all other composite beam stems in 4 separate cohorts (Table 4, see Supplementary data). Reasons for revision are shown (Table 5).

**Table 5.** Reasons for revision by stem type. Values are frequency (%) (multiple reasons allowable)

Reason for revision	Total n = 292,987	Exeter (taper-slip) n = 176,189	All other taper-slip n = 69,043	Charnley (composite beam) n = 23,141	All other composite beam n = 24,445	p-value
Aseptic loosening stem	490	169 (0.1)	97 (0.1)	148 (0.6)	76 (0.3)	< 0.001
Aseptic loosening socket	722	395 (0.2)	147 (0.2)	83 (0.4)	97 (0.4)	< 0.001
Dislocation	1140	638 (0.4)	296 (0.4)	108 (0.5)	98 (0.4)	0.02
Stem fracture	50	30 (0.02)	16 (0.02)	2 (0.01)	2 (0.01)	0.3
Infection	921	516 (0.3)	181 (0.3)	125 (0.5)	99 (0.4)	< 0.001
Stem lysis	127	48 (0.03)	28 (0.04)	32 (0.14)	19 (0.08)	< 0.001
Pain	570	285 (0.2)	157 (0.2)	74 (0.3)	54 (0.2)	< 0.001
Peri-prosthetic fracture stem	437	215 (0.1)	192 (0.3)	18 (0.1)	12 (0.05)	< 0.001
Other	1,035	559 (0.3)	270 (0.4)	104 (0.4)	102 (0.4)	< 0.001
Total	5,492 (1.9)	2,855 (1.6)	1,384 (2.0)	694 (3.0)	559 (2.3)	< 0.001

The risk of aseptic loosening and stem lysis was higher for composite beam stems than taper-slip varieties (Table 5), as were the rates of revision for infection. There was a difference in the risk of peri-prosthetic fracture between the most implanted taper-slip stem design (0.1%) and all other taper-slip stems (0.3%), both higher than the composite beam groups, which was statistically significant (p < 0.001, chi-squared test). All other reasons for revision were of similar incidence between the 2 stem designs.

**Figure 2.**
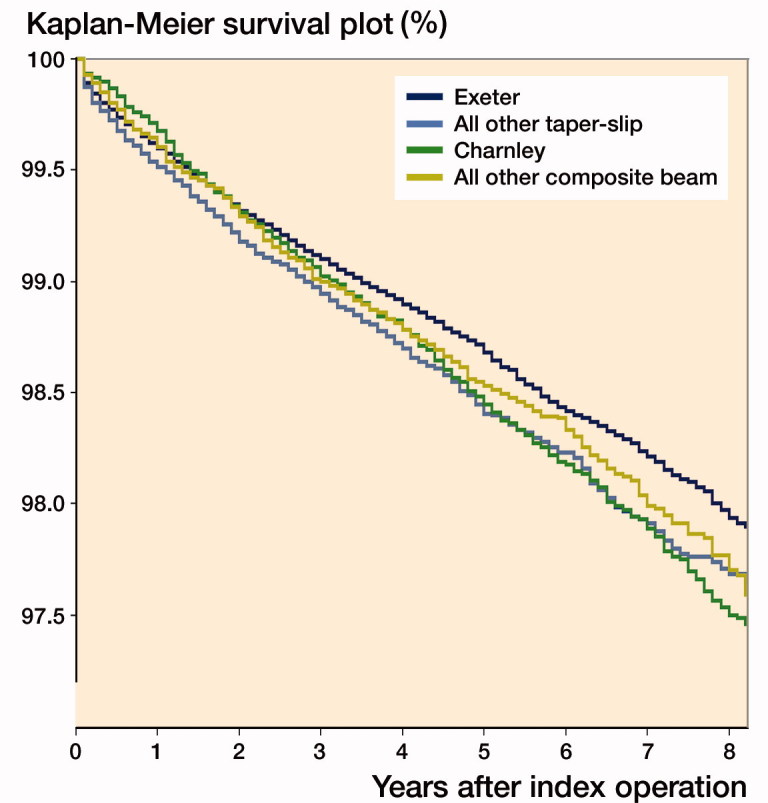
Kaplan–Meier survival curve for most implanted taper-slip, all other taper-slip, most implanted composite beam and all other composite stems. Exeter 97.9% (CI 97.8–98.0), all other taper-slip 97.6% (97.4–97.8), Charnley 97.5% (CI 97.2–97.8), and all other composite beam 97.7% (CI 97.4–98.0) 8-year survival.

When the dataset was further subdivided to assess all 4 groups, however, the survival curves changed, with a superior survival for the most commonly implanted taper-slip stem compared with all other taper-slip (p < 0.001) and most commonly implanted composite beam (p = 0.01), (Figure 2, Table 6).

**Table 6.** Survival rates between groups

Stem type	Kaplan–Meier 8-year survival (95% CI)
Most implanted taper-slip (Exeter)	97.9% (CI 97.8–98.0)
All other taper-slip	97.6% (CI 97.4–97.8)
Most implanted composite beam (Charnley)	97.5% (CI 97.2–97.8)
All other composite beam	97.7% (CI 97.4–98.0)

**Figure 3.**
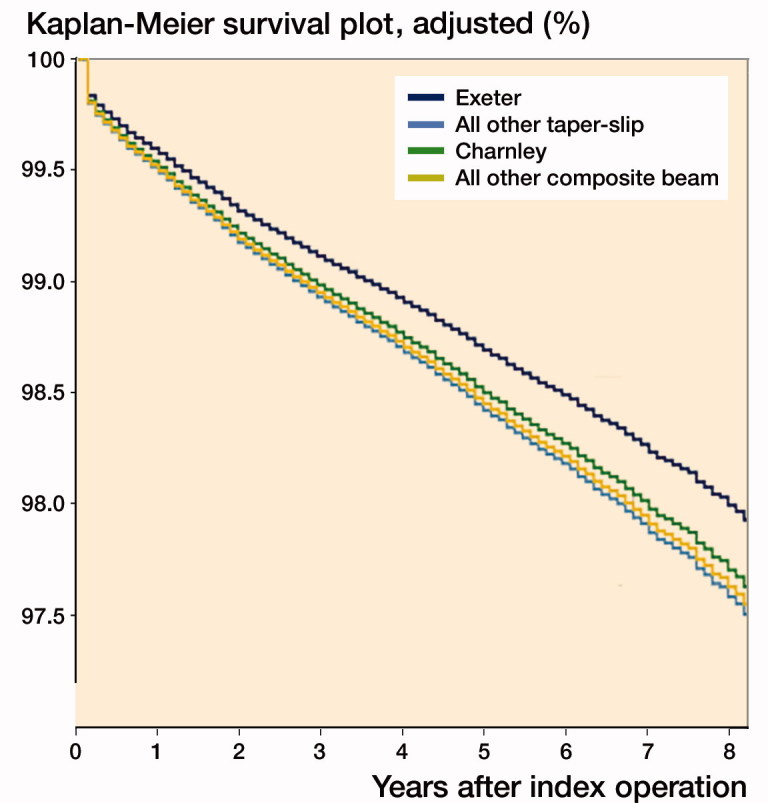
Plot of survival functions for each group when adjusted for confounders using Cox regression.

Finally, in order to adjust for known confounders (age, sex, diagnosis, ASA grade, procedure type, approach, and bearing couple), Cox regression analysis was performed (Table 7, see Supplementary data) and adjusted survival curves plotted (Figure 3), indicating the superior results of the most implanted taper-slip (Exeter) group (all other taper-slip HR 1.2 [CI 1.1–1.3]; Charnley HR 1.2 [CI 1.0–1.3]; other composite beam HR 1.2 [CI 1.1–1.3]). These results remained consistent when taper-slip and composite beam were compared; HR 1.1 (CI 1.0–1.2).

**Table 7.** Hazard ratios (HR), 95% confidence intervals, and significance levels for the Cox regression model, adjusted for known confounders. Only cases with no missing values included in the model

Variable (reference variable)	n	HR (95% CI)	p-value
Age at primary		0.98 (0.97–0.98)	< 0.001
Sex			
Male	101,343	Reference	
Female	191,623	0.78 (0.73–0.83)	< 0.001
Stem type			< 0.001
Exeter	176,350	Reference	
All other taper-slip	69,036	1.21 (1.12–1.30)	< 0.001
Charnley	23,140	1.15 (1.03–1.28)	0.01
All other composite beam	24,440	1.19 (1.06–1.33)	0.003
Diagnosis			< 0.001
Osteoarthritis	264,745	Reference	
Other arthritides	4,315	1.23 (0.98–1.54)	0.07
Previous trauma	3,718	1.47 (1.13–1.90)	0.004
Acute trauma	6,890	1.84 (1.52–2.22)	< 0.001
Previous hip surgery	536	1.44 (0.75–2.78)	0.3
DDH/childhood disease	2,846	0.77 (0.56–1.07)	0.1
Avascular head necrosis	7,305	1.44 (1.22–1.70)	< 0.001
Other (including infection)	2,611	1.38 (1.03–1.85)	0.03
ASA grade			< 0.001
1	40,449	Reference	
2	200,973	1.20 (1.09–1.32)	< 0.001
3	49,580	1.32 (1.18–1.49)	< 0.001
4	1,887	1.17 (0.76–1.82)	0.5
5	77	0.001 (0–2.3x10^21^)	0.8
Procedure type			
Routine	275,341	Reference	
Complex	17,625	0.99 (0.76–1.25)	0.9
Approach			0.1
Posterior	137,176	Reference	
Trans gluteal	129,450	0.93 (0.87–1.00)	0.049
Trochanteric osteotomy	2,067	1.14 (0.79–1.64)	0.5
Other	10,020	0.83 (0.67–1.03)	0.09
Missing/unknown	14,253	0.83 (0.64–1.08)	0.2
Bearing			< 0.001
Metal-on-polyethylene	257,685	Reference	
Ceramic-on-ceramic	16,330	0.68 (0.58–0.79)	< 0.001
Ceramic-on-polyethylene	18,562	0.61 (0.52–0.71)	< 0.001
Ceramic-on-metal	389	1.39 (0.74–2.58)	0.3

## Discussion

This is the first study to assess the performance of cemented femoral components over the first 10 years of NJR data. Our analysis on almost 300,000 THAs in the NJR initially showed similar results between taper-slip and composite beam cemented stems, as predicted from theoretical studies (Schmalzried et al. 1992, Alfaro-Adrian et al. 2001). However, closer examination identified clear differences within those groups when the most implanted of each group was separated out, so clearly the performance of an individual stem design cannot be predicted by a simple categorisation between taper-slip and composite beam.

A study using the Finnish Arthroplasty Register compared the outcomes of the 12 most popular cemented stem designs over a 25-year period. Both the Exeter and Muller straight stem achieved greater than 90% survivorship at 15 years with aseptic loosening as an endpoint (Makela et al. 2008). This again suggests that good results, in terms of survivorship, are possible when composite beam and taper-slip stems are used.

2 randomised trials have been performed comparing stems with different design philosophies. Lachiewicz et al. (2008) enrolled 201 patients (219 hips) and found no differences at 5 years comparing taper-slip and a roughened pre-coat stem in terms of revision for loosening or failure. Jayasuriya et al. (2013) compared a composite beam design (Charnley) with a double-tapered (Exeter) and triple-tapered (C-stem) design. At the 2-year review of 120 patients, no difference in bone remodelling or outcomes between the 3 groups was found.

Numerous cohort (Van Eynde et al. 2010, Broden et al. 2015), case control (Sarvilinna et al. 2005), randomised trials (Lachiewicz et al. 2008) and registry studies (Hailer et al. 2010, Thien et al. 2014) have compared revision rates and peri-prosthetic fracture rates for cemented and uncemented components and have compared peri-prosthetic fracture rates by cemented fixation type. Overall the risk of peri-prosthetic fracture is higher with uncemented stems. In a study of 437,629 patients in the Nordic Arthroplasty Register Association the relative risk for peri-prosthetic fracture in the uncemented group was 8.7 with the risk increasing with increasing age (Thien et al. 2014). Amongst cemented stem designs there is evidence that peri-prosthetic fracture rates are higher in those with a polished tapered stem after hip fracture (Sarvilinna et al. 2005). Thien et al. (2014) in a registry analysis revealed a higher peri-prosthetic fracture risk for a polished tapered stem when compared with a composite beam counterpart. We confirmed that there is a statistically significant difference in peri-prosthetic fracture risk between taper-slip and composite beam stems but this risk is offset by the decreased risk for revision for other indications.

Harris (1998), an advocate of roughened pre-coated stems, reviewed the results of various stem designs and postulated that roughening per se was not deleterious due to the multiple series and designs demonstrating good outcomes. He made the point that specific stem geometry issues may lead to poorer results with some designs more than others. We did not separate the results of different brands of stems that function as composite beam devices but it is worth noting that, even within a single brand, differences in results have been described that have their origins in modifications to the shape and surface finish of the implant (Dall et al. 1993).

Polished tapered stems, be they double- or triple-tapered, have demonstrated excellent long-term results due to their taper-slip geometry and mode of action. The Exeter stem, the most implanted stem identified in the series described, is a polished double-tapered design, earlier iterations of which have shown excellent results at up to 17 years follow-up and beyond in both the design centre (Carrington et al. 2009, Petheram et al. 2016) and independent units (Hook et al. 2006, Young et al. 2009). These results have held true in both the general population and those under 50 years old at the time of surgery. Other designs of collarless, polished, tapered stems also have good published results in the literature (Purbach et al. 2013, Junnila et al. 2016) but we have identified in this registry analysis that, overall, the results of the Exeter stem were statistically significantly better than those of other stems combined. This may be due to some poorly performing stems included in this group, but individual brand comparisons were beyond the scope of this study. The results for almost all indications for revision were improved when the market-leading stem was implanted.

Whilst individual studies are useful, the use of registry data has been suggested as a more powerful tool in measuring outcomes for the generalist/non-specialist (Palan et al. 2016). Our study highlights the difference between brands of implant that are assumed to function with the same design philosophy, although is limited by the fact that more detailed, individual brand-specific analysis was beyond the scope of this study.

Large registry studies are able to detect small differences in outcome, although the difference between statistical and clinical significance should be considered, as well as the potential effect of bias (Whitehouse et al. 2014). Although the differences between the groups were small in our study, a difference of 0.5% at 8 years may be clinically significant when attempting to maximise the effectiveness of this highly successful procedure, and highlights that not all stems of a similar philosophy behave in exactly the same manner.

Limitations of our study include the small number of data entries submitted to the NJR in the early years of its existence and the fact that some centres had poor submission compliance data submission, potentially skewing results. Similarly, the revision rates may be higher than reported due to unreliable NJR compliance with data submission at revision surgery (Porter 2017). This is unlikely to skew the findings if the failure to report was equivalent across all stem designs. Residual confounding may also remain due to the limitations of data capture within the NJR (e.g., the use of the Charlson index for comorbidities would be preferential to ASA grade but is not part of the minimum dataset) or inclusion in the analysis (e.g., surgeon experience or Trust preference may dictate which implant is used).

In summary, this large registry review study showed a significant survival advantage of the most popular taper-slip design over all other groups of patients. Future research efforts should focus on brand/design comparison rather than comparing outcomes in different fixation philosophies as this provides more accurate data and results as demonstrated in this paper. Even these comparisons may be skewed by other confounders relevant only at brand level (Junnila et al. 2016).

## Supplementary Material

Supplemental Material

## Supplementary data

Tables 2–4 and 7 with descriptive statistics and results from the Cox regression analysis, and NJR disclaimer are available as supplementary data in the online version of this article, http://dx.doi.org/ 10.1080/17453674.2019.1582680

## Disclaimer

The Healthcare Quality Improvement Partnership (“HQIP”) and/or the National Joint Registry (“NJR”) take no responsibility for the accuracy, currency, reliability, and correctness of any data used or referred to in this report, nor for the accuracy, currency, reliability, and correctness of links or references to other information sources and disclaims all warranties in relation to such data, links, and references to the maximum extent permitted by legislation.

HQIP and NJR shall have no liability (including but not limited to liability by reason of negligence) for any loss, damage, cost, or expense incurred or arising by reason of any person using or relying on the data within this report and whether caused by reason of any error, omission, or misinterpretation in the report or otherwise. This report is not to be taken as advice. Third parties using or relying on the data in this report do so at their own risk and will be responsible for making their own assessment and should verify all relevant representations, statements, and information with their own professional advisers.
