# Supplementary material for: Not all cemented hips are the same: a register-based (NJR) comparison of taper-slip and composite beam femoral stems
Source: Acta Orthop. 2019 Mar 6;90(3):214–9. doi: 10.1080/17453674.2019.1582680 (PMC6534220; doi:10.1080/17453674.2019.1582680)
Supplement: Supplemental Material [file IORT_A_1582680_SM2404.pdf]

Supplementary data

Disclaimer

The views expressed represent those of the authors and do not necessarily reflect those of the National Joint Registry Steering Committee or the Health Quality Improvement Partnership (HQIP) who do not vouch for how the information is presented.

The Healthcare Quality Improvement Partnership ("HQIP") and/or the National Joint Registry ("NJR") take no responsibility for the accuracy, currency, reliability, and correctness of any data used or referred to in this report, nor for the accuracy, currency, reliability, and correctness of links or references to other information sources and disclaims all warranties in relation to such data, links, and references to the maximum extent permitted by legislation.

HQIP and NJR shall have no liability (including but not limited to liability by reason of negligence) for any loss, damage, cost, or expense incurred or arising by reason of any person using or relying on the data within this report and whether caused by reason of any error, omission, or misinterpretation in the report or otherwise. This report is not to be taken as advice. Third parties using or relying on the data in this report do so at their own risk and will be responsible for making their own assessment and should verify all relevant representations, statements, and information with their own professional advisers.

Table 2. Stem type and brand frequencies

Stem	Frequency
**Taper-slip**	
Exeter V40	175,472
CPT	29,512
C-Stem Cemented Stem	17,477
C-Stem AMT Cemented Stem	8,994
MS-30	3,415
CPCS	2,468
CPS Plus	2,119
Furlong Cemented Stem	1,877
Taperfit Cemented Stem	1,313
Taperloc Cemented Stem	1,034
Olympia	904
Exeter	886
Ultima TPS Stem	176
Aeon Cemented Stem	261
Profemur Cemented Stem	241
Corail Cemented	135
Edinburgh	117
Total	245,401
**Composite beam**	
Charnley Cemented Stem	22,015
Stanmore Modular Stem	7,016
Muller-Biomet	3,107
Muller Straight Stem	2,481
SP II Cemented Stem	2,372
Elite Plus Cemented Stem	1,719
Omnifit Cemented Stem	1,513
CCA Cemented Stem	1,302
Charnley Modular	1,126
P10 Muller	838
Spectron	838
Centrament	671
VerSys Cemented Stem	623
CMK Cemented Stem	561
Excia Cemented	438
Ultima Straight Stem	396
Mem	371
Stanmore Monobloc Stem	237
Bimetric Cemented (Ti)	209
Hi-Nek Cemented Stem	129
Summit Cemented Stem	108
Bimetric Cemented (CoCr)	104
Total	47,586

Table 3. Comparison of taper-slip and composite beam stems

Factor	Stem type Taper-slip	Composite beam	p-value
Number	245,401 (83.8%)	47,586 (16.2%)	n/a
Mean age (range)	71.9 (12–103)	73.6 (15–101)	< 0.001 [a]
Death, n (%)	25,761 (10.5)	8,168 (17.2)	< 0.001 [b]
Revised, n (%)	3,080 (1.3)	810 (1.7)	< 0.001 [b]

[a] ANOVA
[b] χ^2 test

Table 4. Breakdown by stem type. Values are frequency (%) unless otherwise stated

	Taper-slip		Composite beam		p-value	
	Exeter	All other	Charnley	All other	2 groups	4 groups
Number	176,358	69,043	23,141	24,445	N/A	N/A
Age in years (range)	71.8 (12–100)	72.2 (15–103)	72.9 (19–101)	74.3 (15–101)	< 0.001 [a]	< 0.001 [a]
Death	18,234 (10.3)	7,527 (10.9)	4,215 (18.2)	3,953 (16.2)	< 0.001 [b]	< 0.001 [b]
Revised	2,108 (1.2)	972 (1.4)	428 (1.8)	382 (1.6)	< 0.001 [b]	< 0.001 [b]
Sex					< 0.001 [b]	< 0.001 [b]
Male	62,345 (35.4)	23,137 (33.5)	8,174 (35.3)	7,694 (31.5)		
Female	114,012 (64.6)	45,903 (66.5)	14,967 (64.7)	16,750 (68.5)		
ASA Grade					< 0.001 [b]	< 0.001 [b]
1	24,911 (14.1)	8,769 (12.7)	3,443 (14.9)	3,727 (13.6)		
2	120,756 (68.5)	48,327 (70.0)	15,043 (65.0)	16,859 (69.0)		
3	29,577 (16.8)	11,497 (16.7)	4,436 (19.2)	4,077 (16.7)		
4	1,073 (0.6)	436 (0.6)	210 (0.9)	169 (0.7)		
5	41 (0.02)	14 (0.02)	9 (0.04)	13 (0.1)		
Procedure type					< 0.001 [b]	< 0.001 [b]
Routine	168,339 (95.5)	65,662 (95.1)	19,466 (84.1)	21,894 (89.6)		
Complex	8,019 (4.5)	3,381 (4.9)	3,675 (15.9)	2,551 (10.4)		
Diagnosis					< 0.001 [b]	< 0.001 [b]
Osteoarthritis	158,824 (90.1)	62,244 (90.2)	21,341 (92.2)	22,351 (91.4)		
Other arthritides	2,651 (1.5)	943 (1.4)	403 (1.7)	319 (1.3)		
Previous trauma	2,313 (1.3)	876 (1.3)	212 (0.9)	317 (1.3)		
Acute trauma	4,489 (2.5)	1,579 (2.3)	229 (1.0)	596 (2.4)		
Previous hip surgery	328 (0.2)	172 (0.2)	13 (0.1)	23 (0.1)		
Childhood disease	1,807 (1.0)	820 (1.2)	103 (0.4)	116 (0.5)		
Avascular head necrosis	4,293 (2.4)	1,871 (2.7)	618 (2.7)	524 (2.1)		
Other (including infection)	1,653 (0.9)	538 (0.8)	222 (1.0)	199 (0.8)		
Approach					< 0.001 [b]	< 0.001 [b]
Transgluteal	73,779 (41.0)	26,466 (38.3)	16,638 (71.9)	14,028 (57.4)		
Posterior	92,779 (52.6)	36,047 (52.2)	1,442 (6.2)	6,906 (28.3)		
Trochanteric osteotomy	91 (0.1)	1,272 (1.8)	680 (2.9)	24 (0.1)		
Other	5,225 (3.0)	2,721 (3.9)	914 (3.9)	1,161 (4.7)		
Missing	5,934 (3.4)	2,527 (3.7)	3,467 (15.0)	2,326 (9.5)		
Bearing						
Metal-on-polyethylene	155,028 (87.9)	58,315 (84.5)	22,728 (98.2)	21,633 (88.5)		
Ceramic-on-ceramic	12,192 (6.9)	3,392 (4.9)	252 (1.1)	494 (2.0)		
Ceramic-on-polyethylene	8,853 (5.0)	7,251 (10.5)	160 (0.7)	2,300 (9.4)		
Ceramic-on-metal	285 (0.2)	85 (0.1)	1 (0.004)	18 (0.1)	< 0.001 [b]	< 0.001 [b]

[a] ANOVA
[b] χ^2 test

Table 7. Hazard ratios (HR), 95% confidence intervals, and significance levels for the Cox regression model, adjusted for known confounders. Only cases with no missing values included in the model

Variable (reference variable)	n	HR (95% CI)	p-value
Age at primary		0.98 (0.97–0.98)	< 0.001
Sex			
Male	101,343	Reference	
Female	191,623	0.78 (0.73–0.83)	< 0.001
Stem type			< 0.001
Exeter	176,350	Reference	
All other taper-slip	69,036	1.21 (1.12–1.30)	< 0.001
Charnley	23,140	1.15 (1.03–1.28)	0.01
All other composite beam	24,440	1.19 (1.06–1.33)	0.003
Diagnosis			< 0.001
Osteoarthritis	264,745	Reference	
Other arthritides	4,315	1.23 (0.98–1.54)	0.07
Previous trauma	3,718	1.47 (1.13–1.90)	0.004
Acute trauma	6,890	1.84 (1.52–2.22)	< 0.001
Previous hip surgery	536	1.44 (0.75–2.78)	0.3
DDH/childhood disease	2,846	0.77 (0.56–1.07)	0.1
Avascular head necrosis	7,305	1.44 (1.22–1.70)	< 0.001
Other (including infection)	2,611	1.38 (1.03–1.85)	0.03
ASA grade			< 0.001
1	40,449	Reference	
2	200,973	1.20 (1.09–1.32)	< 0.001
3	49,580	1.32 (1.18–1.49)	< 0.001
4	1,887	1.17 (0.76–1.82)	0.5
5	77	0.001 (0–2.3×10^{21})	0.8
Procedure type			
Routine	275,341	Reference	
Complex	17,625	0.99 (0.76–1.25)	0.9
Approach			0.1
Posterior	137,176	Reference	
Trans gluteal	129,450	0.93 (0.87–1.00)	0.049
Trochanteric osteotomy	2,067	1.14 (0.79–1.64)	0.5
Other	10,020	0.83 (0.67–1.03)	0.09
Missing/unknown	14,253	0.83 (0.64–1.08)	0.2
Bearing			< 0.001
Metal-on-polyethylene	257,685	Reference	
Ceramic-on-ceramic	16,330	0.68 (0.58–0.79)	< 0.001
Ceramic-on-polyethylene	18,562	0.61 (0.52–0.71)	< 0.001
Ceramic-on-metal	389	1.39 (0.74–2.58)	0.3
